# Preparation and Characterization of Thermoelectric PEDOT/Te Nanorod Array Composite Films

**DOI:** 10.3390/ma15010148

**Published:** 2021-12-25

**Authors:** Hong-Ju Ahn, Seil Kim, Kwang Ho Kim, Joo-Yul Lee

**Affiliations:** 1 Electrochemistry Department, Korea Institute of Materials Science, Changwon 51508, Korea; ghdwn9202@kims.re.kr; 2 School of Materials Science and Engineering, Pusan National University, Busan 46241, Korea

**Keywords:** thermoelectric material, galvanic displacement reaction, electrochemical polymerization, tellurium, PEDOT, inorganic-organic hybrid material

## Abstract

In this study, we prepared Te nanorod arrays via a galvanic displacement reaction (GDR) on a Si wafer, and their composite with poly(3,4-ethylenedioxythiophene) (PEDOT) were successfully synthesized by electrochemical polymerization with lithium perchlorate (LiClO_4_) as a counter ion. The thermoelectric performance of the composite film was optimized by adjusting the polymerization time. As a result, a maximum power factor (PF) of 235 µW/mK^2^ was obtained from a PEDOT/Te composite film electrochemically polymerized for 15 s at room temperature, which was 11.7 times higher than that of the PEDOT film, corresponding to a Seebeck coefficient (*S*) of 290 µV/K and electrical conductivity (*σ*) of 28 S/cm. This outstanding PF was due to the enhanced interface interaction and carrier energy filtering effect at the interfacial potential barrier between the PEDOT and Te nanorods. This study demonstrates that the combination of an inorganic Te nanorod array with electrodeposited PEDOT is a promising strategy for developing high-performance thermoelectric materials.

## 1. Introduction

Most of the world’s power is produced by combustion engines that use fossil fuels such as coal, oil, and natural gas as an energy source. However, the efficiency of an internal combustion engine is only 30–35%, with approximately 40% of the energy being emitted to the environment as exhaust [1]. To improve engine efficiency, it is necessary to develop a method for the use of waste heat generated from heat engines. Thermoelectric generators are recognized as promising green energy technologies for converting waste heat or natural heat into electricity and vice versa, which are generally in the form of solid-state devices that contain no moving parts, and can operate without maintenance for a long time [2].

The thermoelectric performance is defined in terms of the dimensionless figure-of-merit *ZT*, *S*^2^σT/κ, where *S* is the Seebeck coefficient, σ is the electrical conductivity, κ is the thermal conductivity, and T is the absolute temperature. The numerator of Z, *S*^2^σ, is called the power factor (PF), which is used to estimate and compare performance of film-type thermoelectric materials [3]. Inorganic materials such as bismuth telluride (Bi_2_Te_3_) and its alloys exhibit the best thermoelectric performance near room temperature. However, these materials are typically expensive, less abundant, and inherently brittle, which restricts their usage for a wide range and large-scale applications [4]. To overcome these problems, organic conducting polymers such as poly(3,4-ethylenedioxythiophene) (PEDOT) and polyaniline (PANI) are considered as thermoelectric materials because they have numerous advantages, including low cost, low density, high flexibility, and simple process, allowing for a wide range of potential applications. Among these organic conducting polymers, PEDOT is the most remarkable one owing to its high electrical conductivity, optical transparency, flexibility, and environmental stability [5].

Nevertheless, the PF of PEDOT is low compared to that of the inorganic materials, because the conventional techniques to improve the PF lead to a shift of Fermi energy levels toward the edge of the conduction band, resulting in a reduction in *S* [6]. Recently, inorganic-organic hybrid materials have received great attention worldwide owing to their potential to improve the thermoelectric performance by utilizing the organic materials with low thermal conductivity and the inorganic materials with high *S* (e.g., Tellurium (Te) [7], SnSe [8], Bi_2_Te_3_ [9], and Sb_2_Te_3_ [10]). The interaction between the inorganic and polymer components in the hybrid materials results in a decoupling of the thermoelectric parameters, leading to significantly improved PF, compared to using the individual components alone [11]. In particular, Te has been recognized as an efficient inorganic filler to enhance the *S* of conducting polymer-based composites because it possesses excellent *S* (~400 µV/K) [12]. Song et al. prepared a PEDOT:poly(styrenesulfonate) (PEDOT:PSS)/PEDOT:PSS-functionalized Te composite film using in situ synthesis followed by a vacuum-assisted filtration process [13]. Meng et al. fabricated a PEDOT:PSS-coated Te nanorod/PEDOT:PSS composite film using a drop-casting technique, and H_2_SO_4_ treatment was employed to improve the thermoelectric properties [14]. Karalis et al. demonstrated a water-based scalable synthetic method for the fabrication of a thermoelectric hybrid ink consisting of Te nanowires and PEDOT:PSS [15]. These methods usually use solution-based synthesis techniques such as the solvothermal method, polyol process, and hydrothermal method to synthesize Te nanostructures, which produce suspended nanostructures that require further processing when used to fabricate nanodevices [16].

Te nanostructures, including nanowires, nanorods, and nanotubes, can be easily synthesized using the galvanic displacement reaction (GDR), which is a spontaneous electrochemical reaction driven by the difference in redox potentials between a sacrificial material and noble metal ions in solution [17]. This technique has advantages such as cost effectiveness, high throughput, and ability to control the crystal structure, morphology, and crystallinity of the material. The ideal design of a composite material involves an individual inorganic filler that is homogeneously dispersed in a polymeric matrix. However, it is difficult to incorporate Te nanostructures into a polymeric matrix because of the incompatibility between the two components. Recently, a hybrid composite strategy with a layered structure has been considered as a potential technique, which is expected to generate a carrier energy filtering effect at the interface in an inorganic-organic hybrid structure, resulting in enhanced *S* without significant reduction in *σ* [18]. Ju et al. prepared a PEDOT:PSS-coated Te-substituted SnSe nanosheet/PEDOT multilayer thin film consisting of three repeated stacking layers, and the maximum PF of the multilayer film reached 222 µW/mK^2^ [19]. Liang et al. synthesized a PEDOT:PSS/Te double-layer thin film with a *S* of 656 µV/K and PF of 126.2 µW/mK^2^ at 70 °C [20].

In this study, organic-inorganic TE composite films with layered structures based on galvanically displaced Te nanorod array films and electrochemically polymerized PEDOT were prepared. The inorganic nanostructure with one-dimensionally aligned can allow for effective thermal and electrical energy transfer with low energy loss as forming the composite structure with the conducting polymer, compared to that of randomly dispersed structures. Furthermore, the electrochemical polymerization technique has many advantages, including scalability, fast reaction speed, no requirements for further purification steps, and possibility of tuning the properties of the coated polymers [21]. The proposed synthetic technique of composite films has the advantage that the Si wafer used as a sacrificial material for synthesizing Te nanostructures can be used repeatedly until exhausted, and the electro-polymerization technique improves the interaction between the Te nanorod array and PEDOT with a void-less structure. Furthermore, it has the potential to enable site-specific fabrication of a variety of thermoelectric materials with vertically aligned structures, which would provide a large temperature gradient (ΔT). The resulting composite films were characterized, and their thermoelectric properties, such as *S* and PF, were systemically investigated at room temperature (RT).

## 2. Materials and Methods

### 2.1. Materials

P-type boron-doped Si (100) wafers (1–10 Ω cm, 525 μm) were purchased from Shanghai Semiconductor Wafer Corporation (Shanghai, China). 3,4-ethylenedioxythiophene (EDOT, 97%), lithium perchlorate (LiClO_4_, 95%), and acetonitrile (anhydrous, 99.8%) were supplied by Sigma-Aldrich (St Louis, MO, USA). Hydrogen peroxide (H_2_O_2_, 30%) and sulfuric acid (H_2_SO_4_, 98%) were purchased from Daejung Chemical and Metals Co., Ltd., (Siheung, Korea). Hydrofluoric acid (HF, 48–51%) and tellurium dioxide (TeO_2_, 99.995%) were supplied by JT Baker Chemical Co., Ltd., (Phillipsburg, NJ, USA)) and Alfa Aesar Co., Ltd., (Tianjin, China), respectively.

### 2.2. Galvanic Displacement Reaction (GDR)

**Figure 1:**
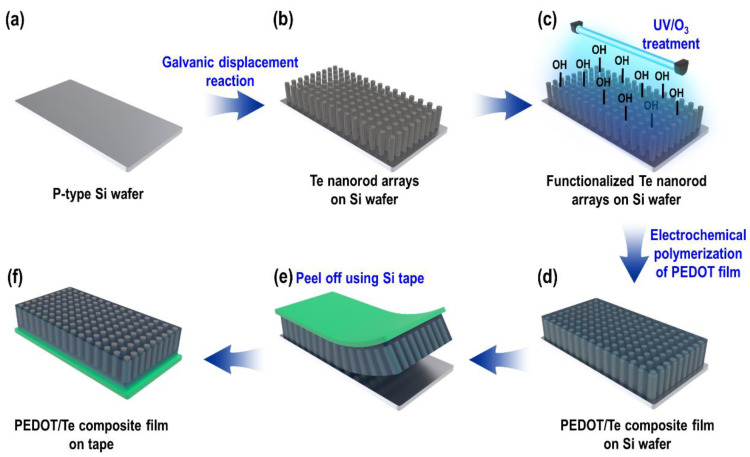
Schematic illustration of poly(3,4-ethylenedioxythiophene) (PEDOT)/Te composite film preparation.

The entire fabrication process for the PEDOT/Te composite film is shown in Figure 1. P-type boron-doped Si wafers were used as the sacrificial substrates for the synthesis of the Te nanostructure. The Si wafer was cleaned using piranha solution containing a mixture of H_2_SO_4_ and H_2_O_2_ (3:1, *v*/*v*) for 30 min at 70 °C, followed by a rinsing 3 times with DI water (Figure 1a). Next, masking was performed on the back side of the Si wafer by covering it with a stop-off lacquer (MICCROSHIELD, Tolber Chemical Division, Pyramid Plastics Inc., Rockford, IL, USA) followed by drying in a vacuum oven for 1 h at 60 °C. Then, the GDR was carried out by immersing the Si wafer in an acidic fluoride bath containing HTeO_2_^+^ ions, which consisted of 1.5 mM TeO_2_, 8.25 M HF, with DI water added to reach a final volume of 300 mL (Figure 1b). The reaction was performed at RT without stirring for 9 hr. After the GDR was completed, the as-synthesized Te nanorod array on the Si wafer was rinsed several times with isopropyl alcohol (IPA) followed by DI water and then blown dry with N_2_ gas.

### 2.3. Synthesis of PEDOT/Te Composite Film

A PEDOT layer was synthesized by electrochemical polymerization, wherein the conducting polymer was deposited from an electrolyte containing the monomer and solvent onto a conducting substrate. Before electrochemical polymerization, ultraviolet light/ozone (UV/O_3_) surface treatment was carried out on the as-synthesized Te nanorod array for 50 s (Model #42, Jelight Co., Ltd., (Irvine, CA, USA) (Figure 1c), to remove the organic impurities and improve the wettability by incorporating oxygen-containing groups on the surface [22,23].

The electrochemical polymerization of PEDOT was performed using a bath containing acetonitrile (100 mL), EDOT (0.01 M), and ClO_4_^−^ (0.1 M) as counter ions (Figure 1d). A three-electrode electrochemical cell system was employed with the galvanically displaced Te nanorod array as the working electrode (cathode, 3 × 3 cm^2^), a Pt mesh electrode as the counter electrode (anode), and an Ag/AgCl electrode as the reference electrode. Chronopotentiometry was carried out at a constant current density of 1 ASD with different PEDOT polymerization times (from 15 to 120 s). The as-synthesized PEDOT/Te composite film was washed several times with DI water and IPA, followed by blowing dry with N_2_ gas. Finally, the composite film was peeled off from the Si wafer using Si adhesive tape (Model #8993K, 3M Company, (Minnesota, MN, USA)).

### 2.4. Characterization

The morphology of the PEDOT/Te composite films was characterized using field emission scanning electron microscopy (FE-SEM, S-4800, Hitachi, Japan). The structural analysis of the PEDOT/Te composite films was performed by an X-ray diffractometer (XRD, D/MAX-2500/PC, Rigaku Co., Tokyo, Japan) using Cu Kα radiation (40 kV, λ = 1.54 Å) with a scan speed of 4°/min in the range of 20°–60°. Raman spectroscopy (High Resolution Raman spectrometer (LabRAM HR-800, HORIBA Jobin Yvon Co., Ltd., Villeneuve d’Ascq, France) measurements were performed in the range of 100–2000 cm^−1^ using an argon ion laser operating at 514 nm. The chemical states of the PEDOT/Te composite films and the work function of each component were characterized by high-resolution X-ray photoelectron spectroscopy (XPS) and ultraviolet photoelectron spectroscopy (UPS, Thermo Fisher Scientific K-Alpha+^TM^, Waltham, MA, USA). The *σ* of the composite films was measured by a standard four-point probe method with a film size of 30 mm × 30 mm at RT. The *S* was measured at RT using a custom-made measurement system that calculated values at temperature differences of less than 2 °C with a two-probe distance of 5 mm, and the heat was generated by applying a voltage using a DC power supply (Agilent 6652 A, Agilent Technologies, Santa Clara, CA, USA). Finally, the PF values of the composite films were calculated using the measured data (σ and *S*).

## 3. Results and Discussion

In this study, Te nanorod arrays were synthesized by GDR, which is generated based on the driving force in the difference of the redox potentials between the solid material and the metal ions in solution. When a Si wafer is immersed in the HF bath containing HTeO_2_^+^ ions, the Si atoms are galvanically displaced by HTeO_2_^+^ ions owing to the difference in redox potential between Si/SiO_2_ (Equation (1)) and HTeO_2_^+^/Te^0^ (Equation (2)), as described in equations blow [24]:

Si^0^ (s) + 2H_2_O (aq) → SiO_2_ (s) + 4H^+^ (aq) + 4e^−^    E^0^ = −0.857 V vs. NHE (1)

SiO_2_ (s) + 6HF (aq) → SiF_6_^2−^ (aq) + 2H^+^ (aq) + 2H_2_O (aq).

HTeO_2_^+^(aq) + 3H^+^ (aq) + 4e^−^ → Te^0^ (s) + 2H_2_O    E^0^ = 0.55 V vs. NHE (2)

where “s” and “aq” indicate the solid and aqueous phases, respectively, and NHE represents the normal hydrogen electrode (E = 0.00 V). At the beginning of the GDR, the SiO_2_ layer was transformed into SiF_6_^2−^ ions in the presence of HF. At the same time, the exposed Si layer reacted with H_2_O, resulting in the formation of a SiO_2_ layer. During this reaction, elemental Te nucleated and grew on the surface of the Si wafer as the HTeO_2_^+^ ions accepting electrons from the SiO_2_ layers.

**Figure 2:**
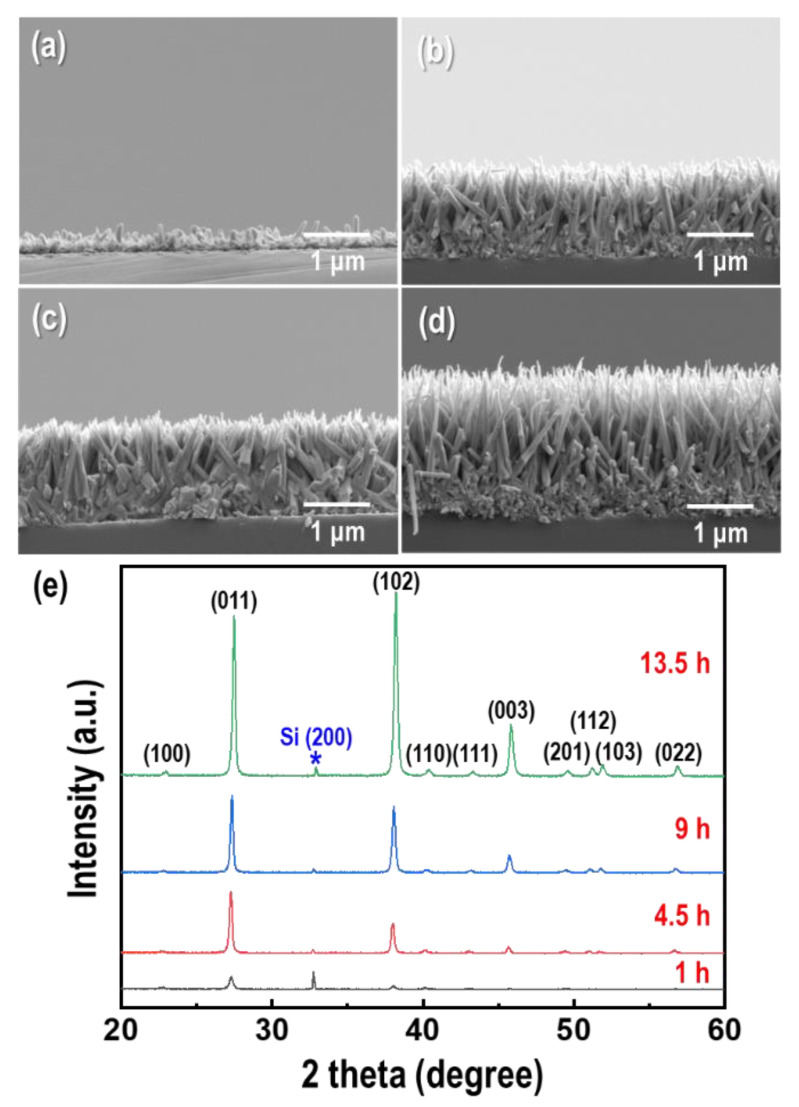
Galvanic displacement reaction (GDR) time dependence of the morphology and dimensions of the Te nanorod arrays. Cross-section field emission scanning electron microscopy (FE-SEM) images of Te nanorod arrays form at (**a**) 1 h, (**b**) 4.5 h, (**c**) 9 h, and (**d**) 13.5 h, respectively. (**e**) Corresponding X-ray diffraction (XRD) patterns of the Te nanorod arrays.

Time-dependent GDR was carried out to investigate the nucleation and growth of Te nanorods on p-type Si wafers, as shown in Figure 2a–d. The concentration of TeO_2_, and HF were fixed at 1.5 mM and 4.5 M, respectively. In the first stage of the GDR, Te nuclei formed on the surface of the Si wafer. Followed the nucleation and growth, a needle-like Te nanorod array with a vertically aligned orientation was formed. It was found that the length of the Te nanorods increased as the GDR time increased, and the growth rate decreased (0.3 μm/h at GDR: 1 h, 0.14 μm/h at GDR: 13.5 h) due to the decrease in the exposed surface of the Si wafer as it was covered by the Te nanorods. The XRD results of the as-synthesized Te nanorod arrays are shown in Figure 2e. All of the diffraction peaks were indexed to the hexagonal structure of Te (JCPDS No. 86-2268) [25], and no other peaks such as tellurium oxide (TeO_2_) existed in the measured product. The increase in the intensity of the (011) peak with increasing GDR time indicated that the crystallinity increased as the GDR proceeded. In particular, the improvement in the intensity of the (003) peak implied a [001] orientation along the c-axis, which was perpendicular to the Si surface [26]. The growth along the [001] direction may be attributed to the highly anisotropic characteristic of Te, which has a hexagonal structure arising from the strong covalent bonding between the neighboring atoms of the same helical chain (intrachain bonding), and weaker interaction between neighboring atoms in adjacent chains (interchain bonding) [27,28].

**Figure 3:**
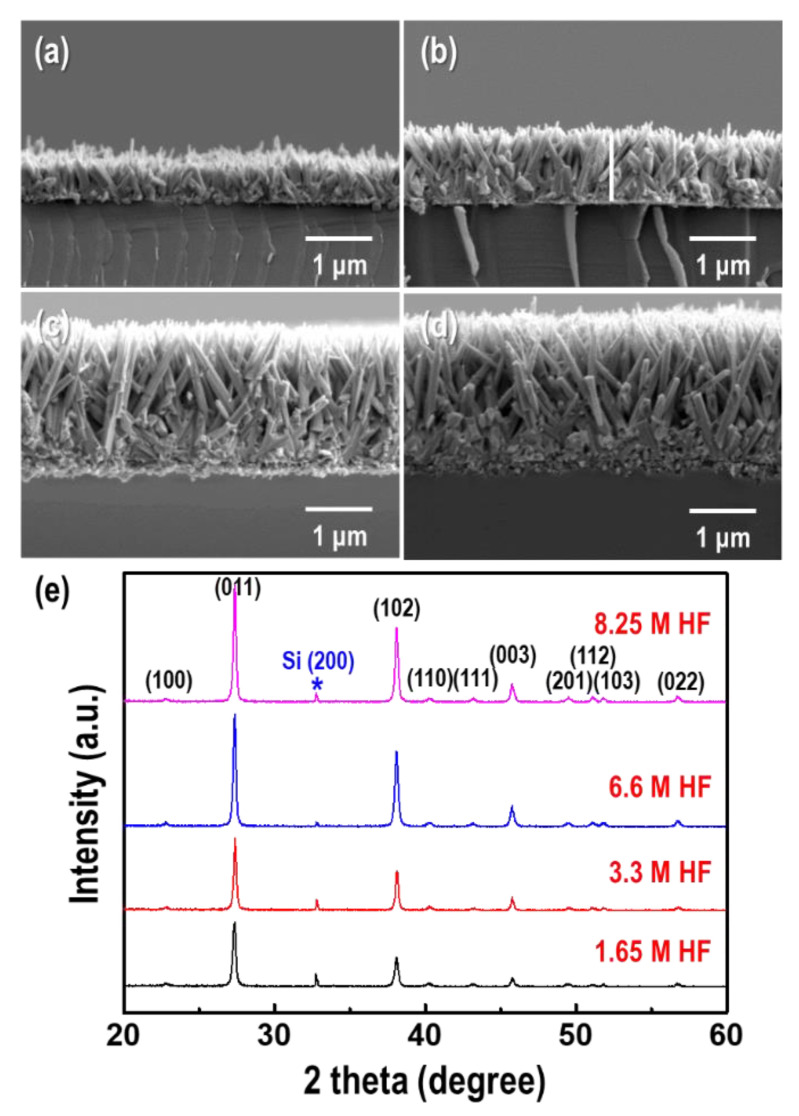
Hydrofluoric acid (HF) concentration dependence of the morphology and dimensions of the Te nanorod arrays. Cross-section FE-SEM images of the Te nanorod arrays formed at HF concentrations of (**a**) 1.65 M, (**b**) 3.3 M, (**c**) 6.6 M. and (**d**) 8.25 M, respectively. (**e**) Corresponding XRD patterns of the Te nanorod arrays. The concentration of TeO_2_ and GDR time were fixed at 1.5 mM and 9 h, respectively.

It is well known that HF is effective in breaking up strong Si-O bonds to form SiF_6_^2−^ ions in acidic solutions. The GDR can be controlled by the dissolution rate of the Si wafer with HF. To investigate the effect of HF on the formation of Te nanorods, different HF concentrations were applied for the GDR, as shown in Figure 3. The concentration of TeO_2_ and GDR time were fixed at 1.5 mM and 9 h, respectively. With increasing HF content (1.65–8.25 M), the length and aspect ratio of the Te nanorods increased by 0.8–2.86 µm and 3.9–16.5, respectively (Figure 3a–d).

This was mainly because the dissolution rate of the Si substrate was proportional to the HF concentration at high-acidic condition, which promoted the GDR by increasing the electron supply. Figure 3e shows the XRD patterns of the Te nanorods synthesized via the GDR at different HF concentrations. The intensities of the (011), (102), and (003) peaks increased with increasing HF content, which implied a [001] orientation along the c-axis.

In this study, the Te nanorod arrays were used as the conducting substrates to synthesize the PEDOT/Te composite film. However, the adhesion of PEDOT is poor because of the hydrophobicity of the surface of the Te nanorod array. The low interfacial adhesion between the PEDOT and Te nanorod arrays will cause cracks, which may reduce the thermoelectric properties of the resulting PEDOT/Te composite films. To decrease the hydrophobicity of the Te nanorod array, surface treatment is required to enhance the adhesive strength between the PEDOT and Te nanorod arrays. Herein, UV/O_3_ treatment was performed on the as-synthesized Te nanorod array prior to the electro-polymerization of PEDOT. This method has many advantages, including being environmentally friendly and low-cost, and can easily induce oxygen-containing groups, which improve the hydrophilicity of the substrate. Te nanorod arrays for UV/O_3_ treatment were prepared by GDR in a bath containing 1.5 mM TeO_2_ and 8.25 M HF. The time for GDR and UV/O_3_ treatment was 9 h and 50 s, respectively.

After UV/O_3_ treatment, the surface of the Te nanorod layer showed hydrophilicity (Figure S1), which was beneficial for the subsequent electro-polymerization process.

**Figure 4:**
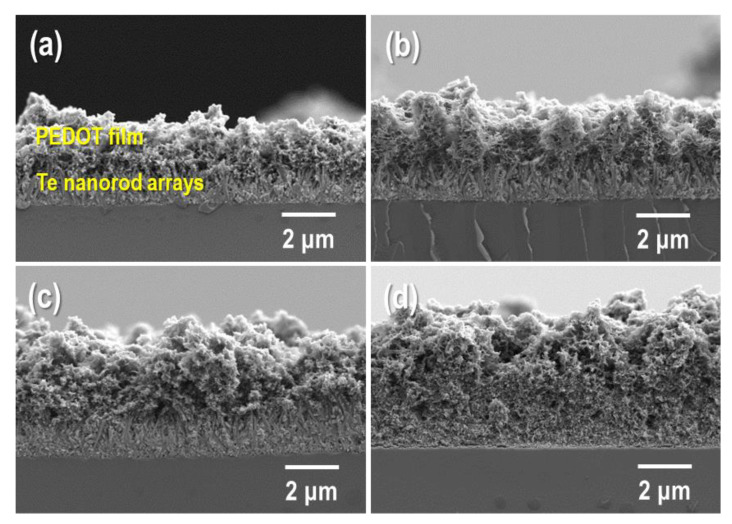
Cross-section FE-SEM images of PEDOT/Te composite films fabricated at the electrochemical polymerization time of (**a**) 15 s, (**b**) 30 s, (**c**) 60 s, and (**d**) 90 s, respectively.

Cross-sectional FE-SEM images of the PEDOT/Te composite films synthesized by electro-polymerization with various polymerization times are shown in Figure 4. When the polymerization time was short (10 s), only a few PEDOT with island structures were deposited in the Te nanorod layer (Figure S2), which disturbed the complexation between PEDOT and the Te nanorod array. As the polymerization time increased to 15 s, a PEDOT layer with a film shape was observed, indicating good deposition of PEDOT onto the Te nanorod array. The thickness of PEDOT increased with increasing polymerization time. For comparison, we also prepared a PEDOT:PSS/Te composite film, in which the Te nanorod array was coated with PEDOT:PSS using a spin-coating method. The resulting composite film had many voids between the PEDOT:PSS and Te nanorod arrays (Figure S3), which caused a decrease in thermoelectric performance.

**Figure 5:**
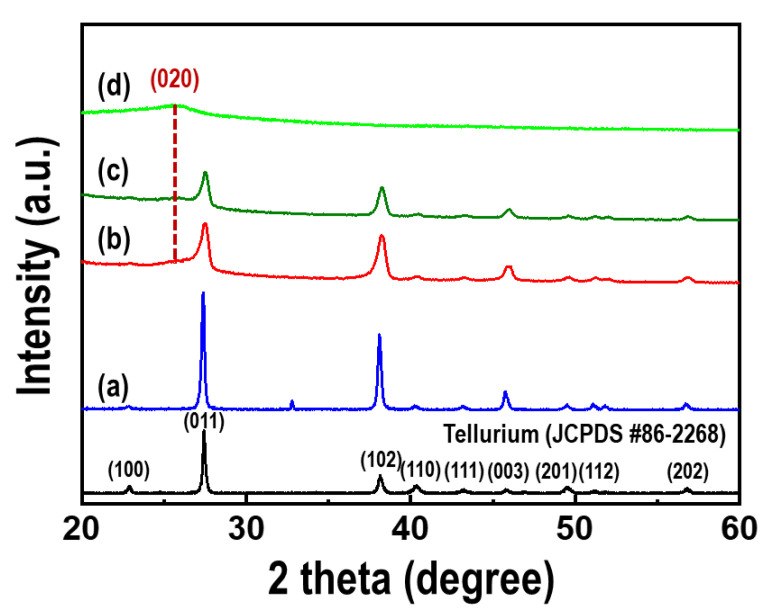
XRD patterns of (**a**) the Te nanorod arrays, PEDOT/Te composite films with polymerization time of (**b**) 15 s and (**c**) 30 s, (**d**) PEDOT film. The black line represents a standard peak of the tellurium (JCPDS No. 86-2268).

The crystal structures of the Te nanorods, PEDOT, and PEDOT/Te composite films were evaluated by XRD, as shown in Figure 5. All the peaks, including the Te nanorod array, agreed with the standard peak of Te (JCPDS No. 86-2268), indicating that the as-synthesized Te nanorods were indexed as hexagonal Te crystal structures. The XRD patterns of the PEDOT/Te composite films are shown in Figure 5b,c. The peak at 2θ = 25.9° corresponded to the (020) plane of PEDOT [29], and both peaks of the PEDOT and Te nanorod arrays were found in the spectra of the PEDOT/Te composite films. When the PEDOT content was high in the composite film, the diffraction peaks of Te were reduced because of the coating PEDOT layer.

**Figure 6:**
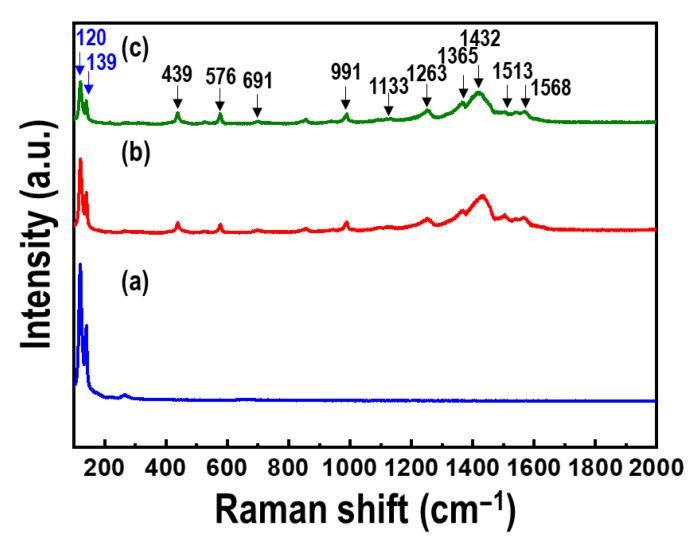
Raman spectra of (**a**) Te nanorod arrays, and PEDOT/Te composite films with polymerization time of (**b**) 15 s and (**c**) 30 s.

**Figure 7:**
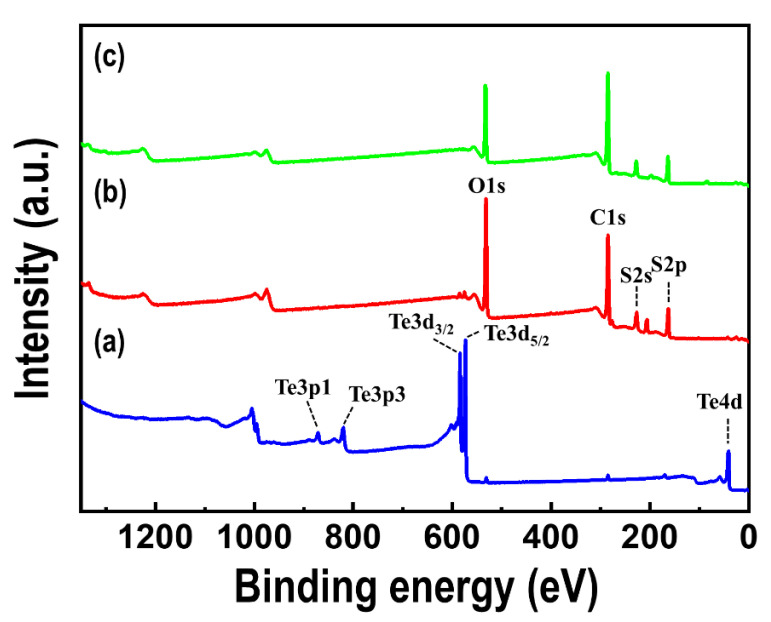
X-ray photoelectron spectroscopy (XPS) spectra of (**a**) Te nanorod array, (**b**) PEDOT/Te composite film with polymerization time of 15 s, and (**c**) PEDOT film.

Figure 6 shows the Raman spectra of the Te nanorod array and PEDOT/Te composite films. Raman bands were observed in all the spectra at 120 and 139 cm^−1^, corresponding to the A_1_ and E_2_ vibration modes of Te, respectively [30,31]. In the case of the PEDOT/Te composite films, bands at 439, 576, and 991 cm^−1^ were observed, which were related to the oxyethylene ring deformation, whereas the bands at 1263 and 1365 cm^−1^ were characteristics of C*_α_*-C*_α_* (inter-ring) stretching and C*_β_*-C*_β_* stretching, respectively [21]. Additionally, the bands at 1432 and 1513 cm^−1^ corresponded to the vibration mode of PEDOT, indicating the C=C symmetric stretching mode and C=C asymmetric stretching mode, respectively. XPS analysis was performed to quantify the elemental surface composition. Figure 7 presents the XPS survey spectra of the Te nanorod array, PEDOT/Te composite film, and PEDOT. For the Te nanorod array, two high-intensity peaks at 585 and 574 eV were observed, corresponding to Te 2d3/2 and Te 3d5/2, respectively [13], and other peaks at 872, 825, and 42 eV, corresponding to Te 3p1, Te 3p3, and Te 4d, respectively [32,33]. After PEDOT polymerization, O, C, and S elements were clearly revealed, originated from the C-O and C-S bonds in PEDOT, and the S2p spectrum of the PEDOT/Te composite film showed a binding energy of 163 eV, corresponding to the S atom of PEDOT. In addition, the polymerization of PEDOT resulted in a reduction of the peak intensity (e.g., Te 3d, Te 4d) from Te and enhancement of the peak intensity (e.g., O1s, C1s, and S2p) from PEDOT, which was consistent with the XRD analysis.

**Figure 8:**
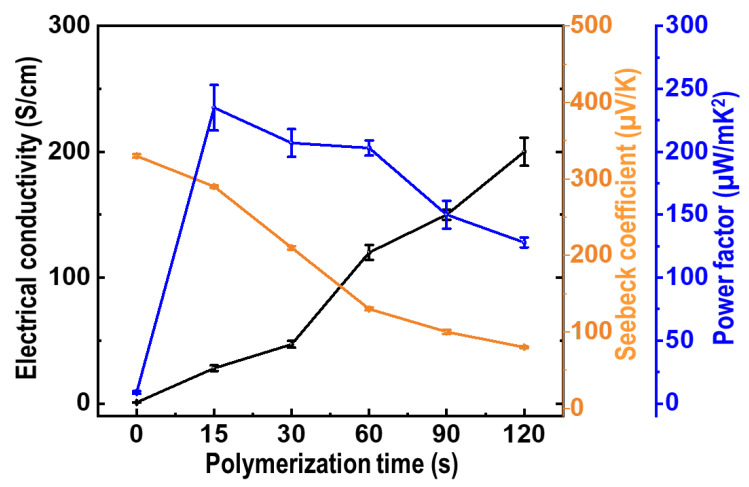
Electrical conductivity (black line), Seebeck coefficient (orange line), and power factor (blue line) of PEDOT/Te composite films as a function of PEDOT polymerization time at room temperature.

Based on the XRD, Raman, and XPS results, the coexistence of Te nanorods and PEDOT components in the composite films was confirmed. The thermoelectric performance of the PEDOT/Te composite films as a function of the PEDOT polymerization time is shown in Figure 8. The *σ* value of the PEDOT/Te composite films increased as the PEDOT polymerization time increased from 15 to 120 s. The high *σ* value was attributed to the fact that the Te nanorod array filled with PEDOT, which improved the electrical conductive pathway in the composite films. Furthermore, the *σ* value of PEDOT was higher than that of the Te nanorod, because its semi-metallic character originated from the high doping concentration of the PEDOT film, which was grown by electro-polymerization with oxidation potential [34]. Moreover, they exhibited p-type semi-metallic behavior and combined into a double-layer structure with a good ohmic contact at the interface between the two phases, which was conducive to the extraction and transmission of high-energy carriers. At a lower PEDOT polymerization time (at 10 s), PEDOT was deposited onto the Te nanorod array and was not well interconnected with the island structure, which caused disconnection of the PEDOT, inhibiting the increase in the *σ* value of the composite film. Upon prolonging the PEDOT deposition time, the *σ* value of the composite film dominated that of PEDOT, and an increase in the *σ* value was observed.

The *S* value was measured as a function of the PEDOT deposition time at room temperature with a custom-made apparatus (Figure 8, orange trace, right scale). The *S* value of the PEDOT/Te composite film was positive, indicating that the resulting films were p-type conducting semiconductors. The *S* value decreased with increasing PEDOT deposition time, from 340 μV/K at 15 s to 80 μV/K at 120 s, whereas the *σ* value showed an opposite trend, which was a typical behavior of thermoelectric materials because of the trade-off relationship between *S* and *σ* [15]. During polymerization of PEDOT onto Te nanorods, the interaction between the π interaction from the PEDOT and van der Waals force from the Te nanorods at the interface of the polymer/Te crystal, lead to enhanced interfacial adhesion between the two phases. At the same time, the carrier energy filtering effect was introduced at the polymer/Te interface, which was beneficial for enhancing the *S* value [35].

PF has been widely used to evaluate the thermoelectric performance of conducting polymer-based materials. The PF of the PEDOT/Te composite films with different PEDOT polymerization times was calculated from the two constituent parameters of the *S* and *σ* values. As a result, the maximum PF (235 µW/mK^2^) was reached for the PEDOT/Te composite film electrodeposited for 15 s, which was approximately 11.75 times higher than that of the PEDOT film (20 µW/mK^2^) and approximately 27 times higher than that of the Te nanorod array (8.7 µW/mK^2^). It was found that there was a synergetic effect between the two components in composite materials. This PF value was higher than that of the previously reported PEDOT:PSS/Te nanostructure-based composites, such as the PEDOT:PSS/Te nanorod composite films (51.4 µW/mK^2^ [13]), PEDOT:PSS/Te nanowire hybrid composites (106 µW/mK^2^ [36]), PEDOT:PSS coated Te nanorod/PEDOT:PSS composite film (141.9 µW/mK^2^ [14]), PEDOT:PSS/Te nanowire-based TE hybrid ink (102.42 µW/mK^2^) [15]), reduced graphene oxide/PEDOT:PSS/Te nanowire hybrid film (143 µW/mK^2^ [37]), and PEDOT:PSS/Te double-layer thin film (126.2 µW/mK^2^ [20]). The outstanding PF of the PEDOT/Te composite film in this study was likely ascribed to the strong interaction between the PEDOT and Te nanorod arrays caused by the carrier energy filtering effect, as well as to the electrochemical polymerization process, which enabled the two components to obtain a strong interfacial adhesion. In particular, the carrier energy filtering approach is an effective method to improve the PF, in which built-in potential barriers block the cold low-energy carriers, while allowing the hot energy carrier to flow, resulting in an increase in *S* at a given carrier concentration [38,39].

**Figure 9:**
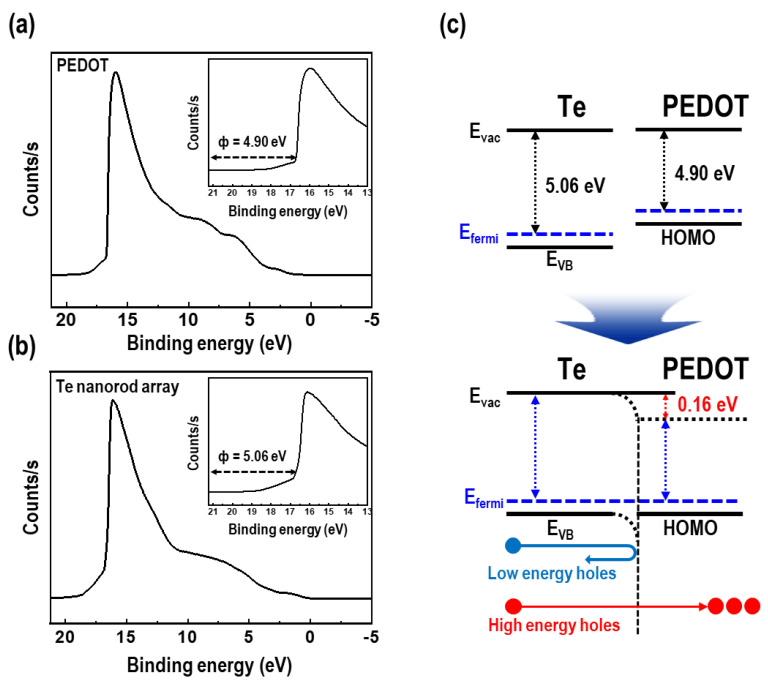
Ultraviolet photoelectron spectroscopy (UPS) spectra of (**a**) PEDOT film and (**b**) Te nanorod array (the insets show the calculated work function). (**c**) Schematic diagram of band-structure at the interfaces between the Te nanorod array and PEDOT.

UPS measurements were performed to examine the carrier energy filtering effect in the PEDOT/Te composite films. The UPS spectra and energy diagrams at the interface between the Te nanorod array and PEDOT are shown in Figure 9. The secondary electron cut-off of PEDOT and Te nanorod arrays were revealed at 16.32 and 16.16 eV, respectively. The work functions of the two materials were calculated using Equation (3) [40]:

Φ = hν − E_cut-off_ (3)

where Φ is the work function, hν is the He (I) excitation energy (21.22 eV), and E_cut-off_ is the secondary electron cut-off. This results showed that the work functions of PEDOT and Te nanorod array was revealed as 4.90 and 5.06 eV, respectively (Figure 9a,b). The work function of the Te nanorod array shown in this study was slightly higher than that of previously reported Te [41,42,43]. This was due to the effect of surface treatment of Te nanorod arrays with UV/O_3_ treatment, which is one of the most common ways to increase the work function, caused by oxidation of the surface [44]. As a result, a potential barrier with a height of ~0.16 eV was formed near the interface between the PEDOT and Te nanorods. A potential barrier height of a few hundreds of meV is recognized as an optimized barrier height to improve the *S* value according to theoretical studies [45]. An effective potential energy barrier was formed between the PEDOT and Te nanorods, filtering holes with energy less than the potential barrier height, while passing holes with high energy at the interface. It is necessary to filter the holes with lower energy because they serve negatively to the *S* value [42], resulting in decreased thermoelectric performance. In other words, the separation of hot and cold energy holes by introducing a potential barrier can help improve the *S* value in organic-inorganic composite systems.

## 4. Conclusions

In this manuscript, a PEDOT/Te composite film with high thermoelectric performance was successfully prepared by the electrochemical polymerization of PEDOT with a Te nanorod array that was synthesized by GDR using a p-type Si wafer as substrate at room temperature. XRD, Raman and XPS characterizations showed the coexistence of Te nanorod and PEDOT constituents in the composite films was confirmed. The thermoelectric properties of the PEDOT/Te composite film were adjusted by changing the polymerization time of PEDOT, resulting in the composite film with a polymerization time of 15 s demonstrating the maximum PF value (235 µW/mK^2^), which was 11.7 times higher than that of the PEDOT film and 27 times higher than that of the Te nanorod arrays. This high PF was due to the carrier energy filtering effect induced by the work function difference between the PEDOT and Te nanorod arrays, and was attributed to the formation of a void-less structure of the composite film via electrochemical polymerization. Especially, double-layer structure of this composite film with two components of p-type semi-metallic behavior plays a critical role in improving interface contact. We conclude that the hybrid composites prepared using Te nanorod array and PEDOT can be promising materials for the production of high performance thin-film thermoelectric devices.

## Data Availability

The data presented in this study are available on request from the corresponding author.

## Supplementary Materials

The following are available online at https://www.mdpi.com/article/10.3390/ma15010148/s1, Figure S1: (a) Contact angle measured without UV/O_3_ exposure to the Te nanorod array. The contact angles measured after the UV/O_3_ exposure with the surface treatment to the Te nanorod arrays for (b) 10, (c) 20, (d) 30, and (e) 50 s, Figure S2: Cross-section field emission scanning electron microscopy (FE-SEM) image of PEDOT/Te composite film synthesized by electrochemical polymerization. The polymerization time was 10 s, Figure S3: Cross-section FE-SEM images of (a) PEDOT/Te composite film synthesized by electrochemical polymerization and (b) PEDOT:PSS/Te composite film synthesized via spin-coating method.
