# Supplementary material for: Preparation and Characterization of Thermoelectric PEDOT/Te Nanorod Array Composite Films
Source: Materials (Basel). 2021 Dec 25;15(1):148. doi: 10.3390/ma15010148 (PMC8745889; doi:10.3390/ma15010148)
Supplement: Supplementary file 1 [file materials-15-00148-s001.zip › materials-1472702-supplementary.pdf]

materials

Supplementary Materials

Preparation and Characterization of Thermoelectric PEDOT/Te Nanorod Array Composite Films

Hong-Ju Ahn [1,2], Seil Kim [1,*], Kwang Ho Kim [2,*] and Joo-Yul Lee [1,*]

[1] Electrochemistry Department, Korea Institute of Materials Science, Changwon 51508, Korea; ghdwn9202@kims.re.kr
[2] School of Materials Science and Engineering, Pusan National University, Busan 46241, Korea
* Correspondence: sikim@kims.re.kr (S.K.); kwhokim@pusan.ac.kr (K.H.K.); leeact@kims.re.kr (J.-Y.L.)

Citation: Ahn, H.-J.; Kim, S.; Kim, K.H.; Lee, J.-Y. Preparation and Characterization of Thermoelectric PEDOT/Te Nanorod Array Composite Films. *Materials* **2022**, *15*, 148. https://doi.org/10.3390/ma15010148

Academic Editor: Denis Music

Received: 4 November 2021
Accepted: 22 December 2021
Published: 25 December 2021

Publisher's Note: MDPI stays neutral with regard to jurisdictional claims in published maps and institutional affiliations.

Copyright: © 2021 by the authors. Licensee MDPI, Basel, Switzerland. This article is an open access article distributed under the terms and conditions of the Creative Commons Attribution (CC BY) license (http://creativecommons.org/licenses/by/4.0/).

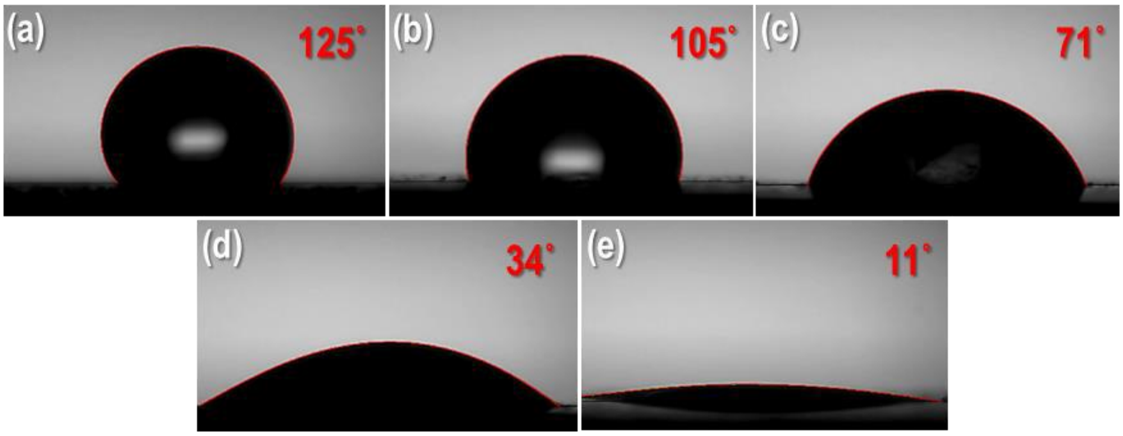

Figure S1. (**a**) Contact angle measured without UV/O_3 exposure to the Te nanorod array. The contact angles measured after the UV/O_3 exposure with the surface treatment to the Te nanorod arrays for (**b**) 10, (**c**) 20, (**d**) 30, and (**e**) 50 s.

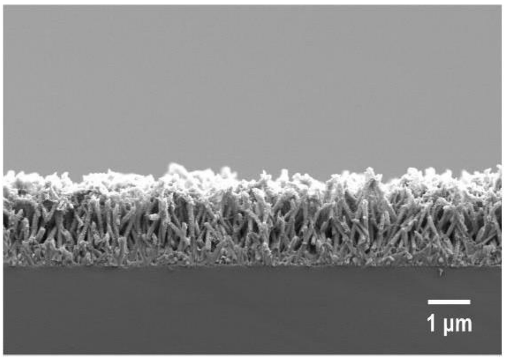

Figure S2. Cross-section field emission scanning electron microscopy (FE-SEM) image of PEDOT/Te composite film synthesized by electrochemical polymerization. The polymerization time was 10 s.

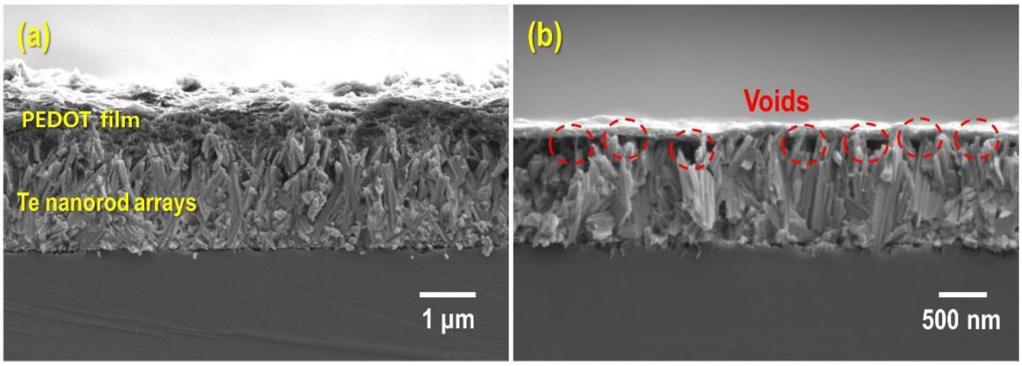

Figure S3. Cross-section FE-SEM images of (**a**) PEDOT/Te composite film synthesized by electrochemical polymerization and (**b**) PEDOT:PSS/Te composite film synthesized via spin-coating method.
